# Supplementary material for: Optimisation of Urine Sample Preparation for Headspace-Solid Phase Microextraction Gas Chromatography-Mass Spectrometry: Altering Sample pH, Sulphuric Acid Concentration and Phase Ratio
Source: Metabolites. 2020 Nov 25;10(12):482. doi: 10.3390/metabo10120482 (PMC7760603; doi:10.3390/metabo10120482)
Supplement: Supplementary file 1 [file metabolites-10-00482-s001.zip › Supplementary Figures.docx]

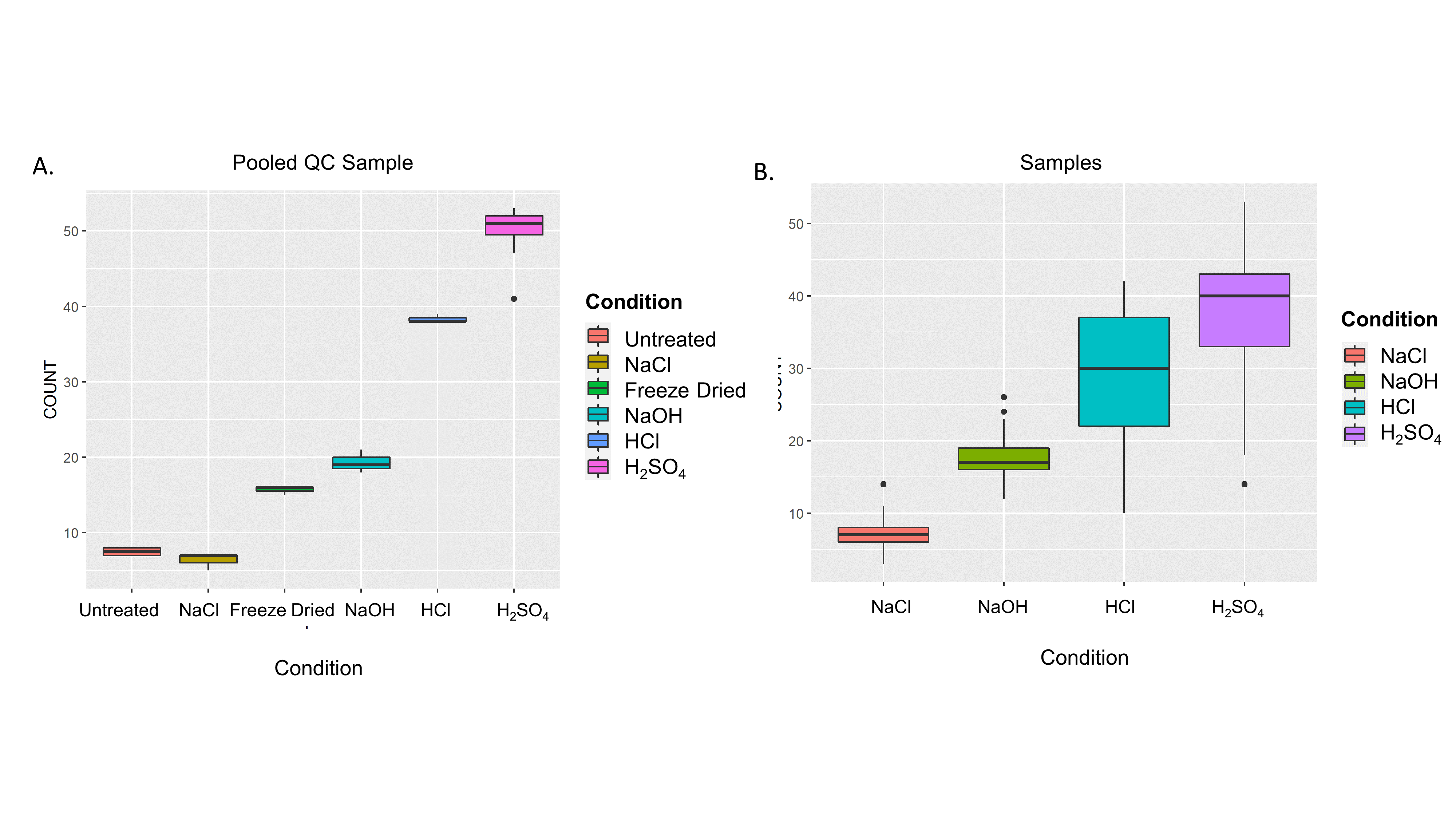


**Figure S1.** Number of VOCs detected when altering pH or ionic strength using saturated NaCl solution. (**a**) Number of VOCs detected in pooled QC samples using different conditions. (**b**) n=26 paired urine samples treated with either saturated NaCl, 5 M NaOH, 5 M HCl or 5 M H2SO4.


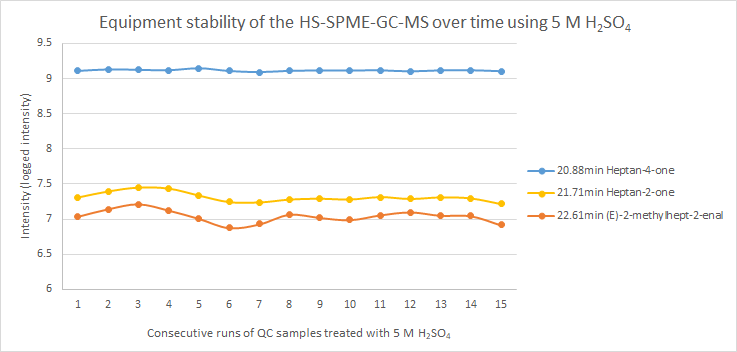


**Figure S2.** Equipment stability of the HS-SPME-GC-MS over time using 5 M H_2_SO_4_. Intensity (logged) of the three most intense VOC peaks detected (Heptan-4-one, Heptan-2-one and (E)-2-methylhept-2-enal) in urine treated with 5 M H_2_SO_4_, found in pooled urine QC samples over 15 days.
