# Supplementary figures and images for: Optimisation of Urine Sample Preparation for Headspace-Solid Phase Microextraction Gas Chromatography-Mass Spectrometry: Altering Sample pH, Sulphuric Acid Concentration and Phase Ratio
Source: Metabolites. 2020 Nov 25;10(12):482. doi: 10.3390/metabo10120482 (PMC7760603; doi:10.3390/metabo10120482)

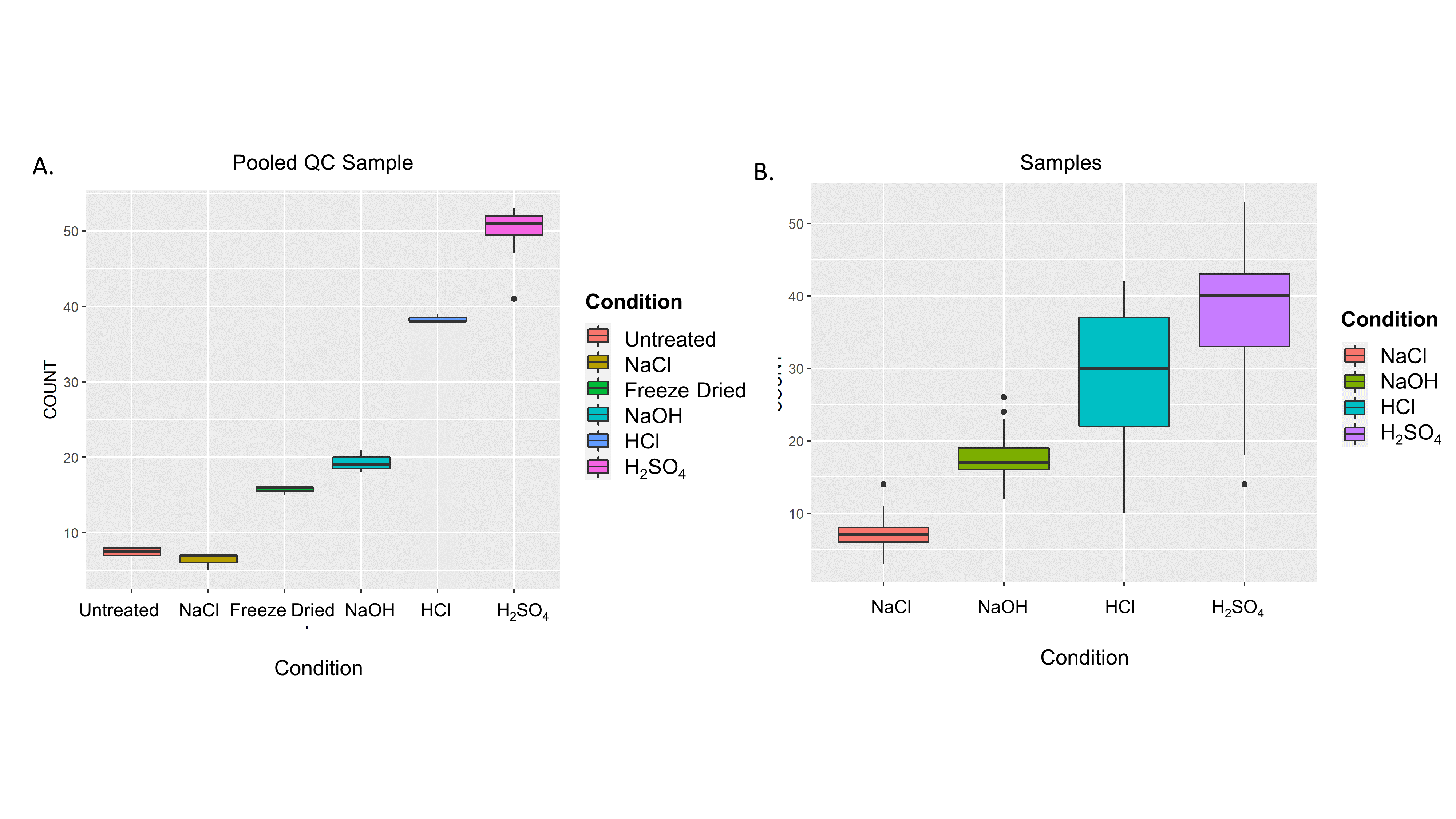

Supplement: Supplementary file 1 [file metabolites-10-00482-s001.zip › Supplementary Figure S1.png]

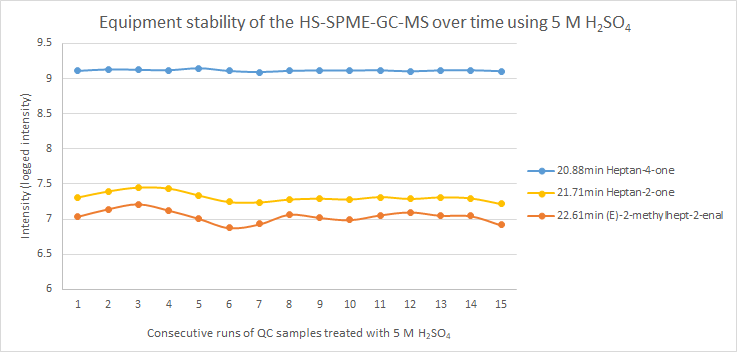

Supplement: Supplementary file 1 [file metabolites-10-00482-s001.zip › Supplementary Figure S2.png]
